# Supplementary material for: Occupational exposure to suicide: A review of research on the experiences of mental health professionals and first responders
Source: PLoS One. 2021 Apr 30;16(4):e0251038. doi: 10.1371/journal.pone.0251038 (PMC8087020; doi:10.1371/journal.pone.0251038)
Supplement: S1 Appendix — (DOCX) [file pone.0251038.s002.docx]

**S1 Appendix Complete research strategy**

“Psychology, Clinical/ or Psychotherapy/ or Psychology/ or Health Personnel/ OR Psychiatry/ (MeSH) AND Suicid* ADJ10 expos* OR Suicid* ADJ10 impact* OR Suicid* ADJ10 bereav* OR Suicid* ADJ10 grie* AND client adj suicide* OR patient adj suicid*” and “emergency responders/ or emergency medical technicians/ or firefighters/ or police/ (MeSH) AND Suicid* ADJ10 expos* OR Suicid* ADJ10 impact* OR Suicid* ADJ10 bereav* OR Suicid* ADJ10 grie*”.
