# Supplementary material for: Occupational exposure to suicide: A review of research on the experiences of mental health professionals and first responders
Source: PLoS One. 2021 Apr 30;16(4):e0251038. doi: 10.1371/journal.pone.0251038 (PMC8087020; doi:10.1371/journal.pone.0251038)
Supplement: Attachment — Submitted filename: Response to reviewers.docx [file pone.0251038.s003.docx]

Thank you very much for your favorable assessment of our manuscript and for

reading it so carefully and making such constructive recommendations. All recommendation were accepted and included on the text. We feel the recommendations have greatly improved the quality and clarity of

our manuscript.

Journal recommendations

We have corrected the style elements in line with PLOS requirements.

Funding information was corrected.

Competing interests was corrected.

Caption for the supporting file was corrected.

Reviewer one

Thank you for your review and suggestions to improve our work.
The biggest issue is the wordiness and size of "table 1. Literature review summaries." Point form rather than sentence form would be more appropriate.

- Table 1 was revised and reworded.

Page 5, line 4, please define "high emotional labor".

– Emotional Labor defined.

Page 7 line 1 and 2 are not necessary about why the spreadsheet was turned into a table.

– Phrase removed.

Page 8, first sentence, 15+5+6 does not equal 25. – We have recounted and noticed that it was one extra paper that was not included in the final version. The numbers were corrected.

Page 14, 6th column under Erlich et al, the authors forgot to replace "commit" suicide with "died by" suicide.

– Replaced.

Page 21, first sentence is confusing about why the analysis wasn't broken down by professional category and whether it was this paper's authors or the authors of the papers they reviewed who did this. It would make sense if it read " The MHPs group includes psychologists, psychiatrists, social workers and nurses."

– We have reworded the phrase in order to make sense that the authors of the cited papers have included all those professionals as a single group during analyses.

Page 22, last paragraph needs cleanup of the text citations.

– The last paragraph was cleaned, and the authors were excluded, only the reference number left.

Page 23, line 6, 7 and 8. What evidence do the authors have that male professionals are less inclined to report emotional reactions? If there is no proof, this clause of the sentence should be more tentative.

-After discussion, we decided to remove the phrase as it was not supported by the data, but a anecdotical conclusion.

Page 25 , last line, "attending suicide scenes have a particular PTSD impact". The verb should be has. Furthermore, I think this is a very important point and should be re-iterated in the conclusion.

-The verb was corrected and a few lines were included to the conclusion highlighting this aspect.

Page 26, line 14 & 15, "All the papers that assessed suicide exposure used web-surveys." I was surprised to find out that most of the methodology of the reviewed studies was web surveys, in the discussion. Perhaps there should be a column in the table 1 for methodological design. A quick count revealed that 17/25 studies were web based. Is this true? This also merits some discussion of the shortcomings of web-based surveys.

- We have counted and confirmed this number. A few lines were written to address the potential issues related to this fact, highlighting the limitations of the use.

Page 28, line 17 should read "usage of the scale can lead to an inflated result of professionals being clinically distressed."

-Phrase corrected.

Page 30, last line. should read "might be reduced".

-Corrected.

Reviewer two

The term "suicide exposure" is convenient but not as telling as "exposure to suicide".

-We have replaced all the entries of “suicide exposure” for “exposure to suicide” on the text.

I don't understand the meaning of "scoping" review

Consider writing in the title "...review of research on the experience of mental health professionals and first responders"

- After discussion we decided to change the tittle. Although we explain that our review fits in the description of a scoping review on the method section.

Was it possible to know differences in the nature of the relationship with the client among mental health professionals, e.g., case manager, prescriber, psychotherapist, etc. It should be important to include.

-We came back to the papers, but they did not describe the nature of relationship of professionals. This may be a limitation on data, and we have addressed this issue on our discussion. Thank you.

I find important the "insight" in the Discussion about lack of theory in "framing" the research. I suggest saying more about what "framing" means and what those two theories say.

-We have written some lines trying to describe how research that used theory framing should work, trying to link exposure to suicide and the suicide risk among professionals.
Thank you for your revision.
